# Loneliness, Anxiety Symptoms, Depressive Symptoms, and Suicidal Ideation in the All of Us Dataset

**DOI:** 10.1001/jamanetworkopen.2026.0596

**Published:** 2026-03-04

**Authors:** Katherine Musacchio Schafer, Jacob Franklin, Peter J. Embí, Colin G. Walsh

**Affiliations:** 1 Department of Biomedical Informatics, Vanderbilt University Medical Center, Nashville, Tennessee; 2 Department of Psychiatry and Behavioral Sciences, Vanderbilt University Medical Center, Nashville, Tennessee; 3 Department of Anesthesiology, Vanderbilt University Medical Center, Nashville, Tennessee; 4 Department of Medicine, Vanderbilt University Medical Center, Nashville, Tennessee

## Abstract

**Importance:**

Although anxiety symptoms and depressive symptoms are linked with increases in suicidal ideation, they leave much of the variance in suicidal ideation unexplained. Loneliness may mediate the links between anxiety symptoms and suicidal ideation as well as depressive symptoms and suicidal ideation.

**Objective:**

To analyze the mediating role of loneliness in the association between anxiety symptoms and suicidal ideation as well as in the association between depressive symptoms and suicidal ideation.

**Design, Setting, and Participants:**

This cross-sectional study used data collected between May 31, 2017, and October 1, 2023, from 62 685 US adults who completed the self-report mental health survey portion of the National Institutes of Health’s All of Us Research Program.

**Main Outcomes and Measures:**

Self-report surveys estimated anxiety symptoms (using the 7-item Generalized Anxiety Disorder scale), depressive symptoms (using the first 8 items of the 9-item Patient Health Questionnaire [PHQ-9]), loneliness (using the UCLA Loneliness Scale), and suicidal ideation (using item 9 of the PHQ-9). Analyses were conducted in August of 2025.

**Results:**

The analytic sample of 62 685 individuals had a mean (SD) age of 61.8 (16.1) years and included 40 749 women (65.0%). Anxiety symptoms (*r* = 0.33; *P* < .001), depressive symptoms (*r* = 0.39; *P* < .001), and loneliness (*r* = 0.31; *P* < .001) correlated with suicidal ideation. When controlling for gender and race and ethnicity, depressive symptoms (*B* = 0.017 [95% CI, 0.017-0.019]), anxiety symptoms (*B* = 0.004 [95% CI, 0.004-0.006]), and loneliness (*B* = 0.007 [95% CI, 0.007-0.008]) accounted for significant variability in suicidal ideation. Loneliness partially mediated the association between anxiety symptoms (average causal mediation effect = 0.01; proportion mediated, 0.25; total association, 0.03; *P* < .001) and suicidal ideation as well as depressive symptoms (average causal mediation effect = 0.003; proportion mediated, 0.10; total association, 0.02; *P* < .001) and suicidal ideation, indicating the associations of anxiety and depressive symptoms with suicidal ideation were in part mediated by loneliness.

**Conclusions and Relevance:**

In this cross-sectional study of 62 685 participants from the All of Us Research Program, loneliness partially mediated the association between anxiety symptoms and suicidal ideation as well as depressive symptoms and suicidal ideation. Targeting and reducing loneliness may present a transdiagnostic approach to arrest the progression from anxiety and depressive symptoms toward suicidal ideation.

## Key Points

**Question:**

Does loneliness mediate the association between anxiety symptoms and suicidal ideation as well as depressive symptoms and suicidal ideation?

**Findings:**

In this cross-sectional study of 62 685 individuals, anxiety symptoms, depressive symptoms, and loneliness were positively associated with suicidal ideation. Loneliness mediated the association between anxiety symptoms and suicidal ideation as well as depressive symptoms and suicidal ideation.

**Meaning:**

This study suggests that anxiety and depressive symptoms on their own are associated with increases in suicidal ideation, yet these associations are partially mediated by loneliness; targeting and reducing loneliness may arrest the progression from depression and anxiety toward suicidal ideation.

## Teaser

This cross-sectional study uses data from the National Institute of Health's All of Us Program to analyze the mediating role of loneliness in the association between anxiety symptoms and suicidal ideation and the association between depressive symptoms and suicidal ideation.

## Introduction

Suicide is a public health concern, with more than 48 000 individuals in the US dying by suicide every year.^1^ Suicidal ideation precedes nearly every suicide death and is deleterious in its own right, associated with increased risk of cognitive deficits,^2^ elevated rates of substance use,^3^ and increased mortality even outside the context of suicide.^4^

As such, numerous studies have centered on identifying correlates of suicidal ideation, 2 of which are anxiety symptoms^5,6,7^ and depressive symptoms.^6,8^ They are among the most commonly studied correlates of suicidal ideation and, while they demonstrate strong associations with suicidal ideation across many samples (eg, veterans,^9^ service members,^10^ inpatients,^11^ outpatients,^12^ and community samples^13^), they leave much of the variance in suicidal ideation unexplained.^8^

Additional constructs are associated with suicidal ideation; among these is loneliness. A recent US Surgeon General report^14^ indicated that individuals in the US are experiencing an “epidemic of loneliness,” and extant literature corroborates this. Rates of loneliness among individuals in the US have increased over the past 5 decades,^15^ and loneliness has been linked with increased risk of suicidal ideation in large and varied samples^16^ as well as theoretical suppositions.^17,18^ However, as is the case with anxiety and depressive symptoms, although loneliness is associated with suicidal ideation, it leaves much of the variance unexplained.

The independent associations of anxiety symptoms, depressive symptoms, and loneliness do not account for the full expression of suicidal ideation. It is possible that these constructs may combine in unique ways to explain suicidal ideation. For example, anxiety and depressive symptoms could be linked with suicidal ideation by way of loneliness, such that the associations of anxiety symptoms and depressive symptoms with suicidal ideation are mediated by loneliness. Anxiety symptoms can turn commonplace experiences—such as social interactions,^19^ test taking,^20^ or interoceptive experiences^21^—into difficult and distressing ones, leading to withdrawal and loneliness, which in turn can lead to suicidal ideation. Depressive symptoms—which often include loss of interest or pleasure in activities—can lead to withdrawal and loneliness, which again can lead to suicidal ideation. Beyond its psychological impacts, the physiological effects of loneliness may be associated with this link as well. A burgeoning literature demonstrates that loneliness modulates neuroinflammation.^22,23,24^ Across the lifespan, higher rates of loneliness are associated with increased neuroinflammatory markers, which have been found to have wide-reaching associations with dementia, depression, and cognitive dysfunction. Centrally relevant to the present work, neuroinflammation has also been linked with suicidal ideation.^25,26,27^ It is possible that loneliness leads to neuroinflammation, which in turn begets an onset or intensification of suicidal ideation.

Such a mediating role of loneliness between anxiety symptoms and suicidal ideation as well as depressive symptoms and suicidal ideation has not been explicitly documented within the literature, to our knowledge. Extant literature could hint at this link. For example, the direct associations of anxiety and depressive symptoms with suicidal ideation are robust and well documented. Among veterans, service members, inpatients, outpatients, and community samples, anxiety and depressive symptoms are strongly linked with elevated rates of suicidal ideation.^9,10,11,12,13^ Anxiety and depressive symptoms may have indirect associations with suicidal ideation, in that anxiety and depressive symptoms are associated with increased loneliness,^28,29,30,31^ which in turn is associated with increased suicidal ideation.^32,33,34^

The objective of the present work was to explicitly investigate the mediating role of loneliness between anxiety symptoms and suicidal ideation as well as depressive symptoms and suicidal ideation using a large diverse sample meant to reflect the diverse nature of the US population—the National Institutes of Health (NIH) All of Us Research Program,^35^ 8th Release, Controlled Tier Dataset. This research program hosts a nationally representative cross-sectional dataset with survey data collected from 633 000 people living in the US. Participants self-select into the dataset with options to complete self-report surveys, share electronic health records, and donate data related to wearable electronic devices. One central mission of the All of Us Research Program is to accelerate health research and medical breakthroughs, in part by conducting replication studies such as the one presented in this article.

We hypothesized that anxiety symptoms, depressive symptoms, loneliness, and suicidal ideation would be significantly and positively correlated. We also hypothesized that, when controlling for gender and race and ethnicity, anxiety symptoms, depressive symptoms, and loneliness would account for significant variance in suicidal ideation. Finally, we hypothesized that loneliness would mediate the association between anxiety symptoms and suicidal ideation as well as depressive symptoms and suicidal ideation.

## Methods

### Data Sources and Population

We performed a cross-sectional analysis using the Controlled Tier Data, Version 8, of the All of Us Research Program. Participants reported on demographic features and mental health survey items. This study was approved by the All of Us Research Program institutional review board. Informed consent was gathered by the All of Us Research Program, which delivered information in writing and via video to potential participants about how the program operates, reasonable expectations, and participants’ rights; the program ensures that people who decide to join do so because it is right for them and the nature of their participation. The results adhere to the Strengthening the Reporting of Observational Studies in Epidemiology (STROBE) reporting guideline.

**Figure 1.**
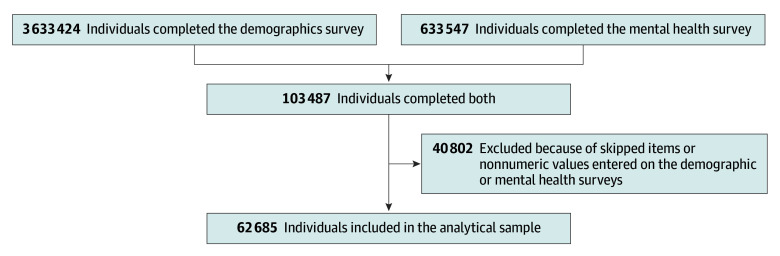
Participant Flowchart From the All of Us Research Program

Figure 1 shows the participant flowchart. From the initial 3 633 424 All of Us participants enrolled from May 31, 2017, to the cutoff for the most recent controlled tier dataset, October 1, 2023, participants were excluded if they did not complete the demographic survey or the mental health surveys. Participants who omitted items on these mental health surveys or entered nonnumeric values were also excluded using listwise deletion.

### Measurements

#### Mental Health Symptoms

In the mental health survey of the All of Us Research Program, participants completed items relating to anxiety symptoms (ie, 7-item Generalized Anxiety Disorder [GAD-7] scale^36^), depressive symptoms (ie, the first 8 items of the 9-item Patient Health Questionnaire [PHQ-9]^37^), loneliness (ie, UCLA Loneliness Scale^38^), and suicidal ideation (ie, item 9 of the PHQ-9). These surveys are criterion standard self-report, psychometrically validated surveys that have been routinely investigated as they related to suicidal ideation.

#### Covariates

Previous research demonstrates differences in anxiety symptoms, depressive symptoms, loneliness, and suicidal ideation based on gender and racial and ethnic groups^39,40,41^; thus, all analyses controlled for gender and race and ethnicity. Race and ethnicity and gender are social constructs with no biological meaning but are associated with differential access to goods, services, and opportunities. Race and ethnicity (American Indian or Alaska Native, Asian, Black or African American, Hispanic or Latino, White, or >1 race or ethnicity) and gender were self-reported by participants. We opted to control only for demographics to avoid overadjustment and preserve the pathways of interest. Although other variables may be associated with the links between anxiety symptoms, depressive symptoms, loneliness, and suicidal ideation, adjusting for them could distort the very associations we aimed to study, which could yield inflated associations between constructs.

### Statistical Analysis

All data analyses were completed in the All of Us Data Workbench using Python, version 3.12.0 (Python Software Foundation). A correlation matrix was constructed to evaluate the cross-sectional independent associations between anxiety symptoms, depressive symptoms, loneliness, and suicidal ideation. A linear regression model controlling for race and ethnicity and gender was conducted to investigate the variance in suicidal ideation explained by anxiety symptoms, depressive symptoms, and loneliness. Finally, 2 mediational models were constructed wherein suicidal ideation was the outcome variable, loneliness scores were the mediators, and anxiety symptoms were the independent variable in the first model and depressive symptoms were the independent variable in the second model. Within mediational models, ACME indicates the average causal mediation effect, ADE indicates the average direct effect, and the proportion mediated quantifies the proportion of the total association from the independent variable with a dependent variable that flows through the mediator (here, loneliness). The total association is the association of the full model. All *P* values were from 2-sided tests and results were deemed statistically significant at *P* < .05.

Sensitivity analyses were conducted to investigate if the mediating roles of loneliness varied between men (eFigure 1 in Supplement 1), women (eFigure 2 in Supplement 1), transgender participants (eFigure 3 in Supplement 1), and nonbinary participants (eFigure 4 in Supplement 1); associations between anxiety symptoms and suicidal ideation as well as depressive symptoms and suicidal ideation by age are reported in eFigure 5 and eFigure 6 in Supplement 1.

## Results

### Demographic Data

Demographic data are displayed in Table 1. This sample comprised 62 685 adult participants (mean [SD] age, 61.8 [16.1] years; 40 749 women [65.0%], 20 891 men [33.4%], 331 nonbinary individuals [0.5%], and 64 transgender individuals [0.1%]; 196 American Indian or Alaska Native [0.3%], 2929 Asian [3.0%], 3436 Black or African American [5.5%], 4600 Hispanic or Latino [7.3%], 50 928 White [81.2%], and 2210 >1 race [3.5%]). The analytic sample was more likely than the overall sample to be White (50 928 [81.2%] vs 1 885 747 [51.9%]), female (40 749 [65.0%] vs 2 194 588 [60.4%]), and not Hispanic or Latino (57 487 [91.7%] vs 3 048 442 [83.9%]). The data related to this sample were cross-sectional; as such, there was no follow-up or follow-up time period. Self-report survey scores reflect a sample with anxiety symptoms (ie, GAD-7 score^36^; mean [SD], 4.6 [4.7]; range 0-21) and depressive symptoms (ie, the first 8 items of the PHQ-9^37^; mean [SD], 4.9 [4.9]; range, 0-24), well below the cutoff of 10. The sample reports loneliness (ie, UCLA Loneliness Scale^38^; mean [SD], 7.6 [5.2]; range, 0-24) exceeding clinical significance of a score over 6. Suicidal ideation (ie, item 9 of the PHQ-9; mean [SD], 0.1 [0.4]; range, 0-3) is consistent with prevalence rates with 6.0% (n = 3752) of the sample endorsing some level of suicidal ideation (score: 0 = 58 844; 1 = 2814; 2 = 533; 3 = 405).

**Table 1.** Demographic Data of the Sample

Variable	No. (%) (N = 62 685)
Gender	
Women	40 749 (65.0)
Men	20 891 (33.4)
Nonbinary	331 (0.5)
Transgender	64 (0.1)
Prefer not to say	21 (0.003)
Skipped	533 (0.9)
Race	
American Indian or Alaska Native	196 (0.3)
Asian	2929 (3.0)
Black or African American	3436 (5.5)
White	50 928 (81.2)
>1 Race	2210 (3.5)
Skipped	4041 (6.4)
Ethnicity	
Not Hispanic or Latino	57 487 (91.7)
Hispanic or Latino	4600 (7.3)
Skipped	509 (0.8)
Age, mean (SD), y	61.8 (16.1)
Anxiety symptom score, mean (SD)^a^	4.6 (4.7)
Depressive symptom score, mean (SD)^a^	4.9 (4.9)
Loneliness score, mean (SD)^a^	7.6 (5.2)
Suicidal ideation score, mean (SD)^a^	0.1 (0.4)

^a^ Anxiety symptoms were assessed using the 7-item Generalized Anxiety Disorder scale, depressive symptoms were assessed using the first 8 items of the 9-item Patient Health Questionnaire (PHQ-9), loneliness was assessed using the UCLA Loneliness Scale, and suicidal ideation was assessed using item 9 of the PHQ-9.

### Correlation Matrix

**Figure 2.**
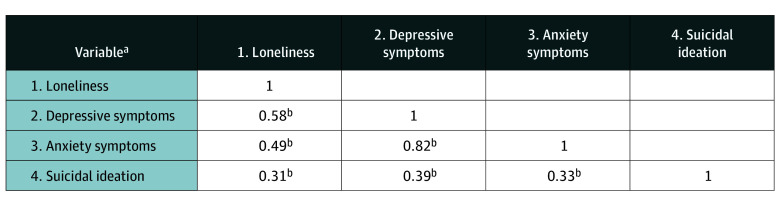
Correlation Matrix for Mental Health Variables ^a^Loneliness was assessed using the UCLA Loneliness Scale, depressive symptoms were assessed using the first 8 items of the 9-item Patient Health Questionnaire (PHQ-9), anxiety symptoms were assessed using the 7-item Generalized Anxiety Disorder scale, and suicidal ideation was assessed using item 9 of the PHQ-9. ^b^*P* < .001.

The correlation matrix is displayed in Figure 2. Anxiety symptoms (*r* = 0.33; *P* < .001), depressive symptoms (*r* = 0.39; *P* < .001), and loneliness (*r* = 0.31; *P* < .001) were positively and significantly associated with suicidal ideation. Depressive symptoms, followed by anxiety symptoms, then by loneliness, held the strongest association with suicidal ideation. The correlation matrix was inspected for collinearity.

### Multiple Linear Regression Models

Table 2 shows the results from the multiple linear regression model, controlling for gender and race and ethnicity. Multiple linear regression models were used to estimate the amount of variance in the dependent variable accounted for by multiple independent variables. In the present study, the amount of variance in suicidal ideation associated with anxiety symptoms, depressive symptoms, and loneliness was assessed. The overall model was significant (*F*_6,62 635_ = 2240; *R*^2^ = 0.18; *P* < .001). Depressive symptoms (*B* = 0.017 [95% CI, 0.017-0.019]), followed by loneliness (*B* = 0.007 [95% CI, 0.007-0.008]), then anxiety symptoms (*B* = 0.004 [95% CI, 0.004-0.006]), accounted for the greatest variance in suicidal ideation. All 3 mental health constructs accounted for significant variance.

**Table 2.** Results From Linear Regression With Suicidal Ideation as the Outcome Variable

Variable	SE	*t* Value	*P* value	*B* Coefficient (95% CI)
Constant	0.007	−15.16	.001	−0.105 (−0.119 to −0.092)
Gender	0.002	17.02	.001	0.027 (0.024 to 0.030)
Race	0.001	0.744	.46	0.001 (−0.001 to 0.003)
Ethnicity	0.006	−1.31	.19	−0.008 (−0.021 to 0.004)
Depressive symptoms^a^	0.001	42.54	.001	0.017 (0.017 to 0.019)
Anxiety symptoms^b^	0.001	8.44	.001	0.004 (0.004 to 0.006)
Loneliness^c^	0.001	22.79	.001	0.007 (0.007 to 0.008)

^a^ Assessed using the first 8 items of the 9-item Patient Health Questionnaire.

^b^ Assessed using the 7-item Generalized Anxiety Disorder scale.

^c^ Assessed using the UCLA Loneliness Scale.

### Mediation Models

**Figure 3.**
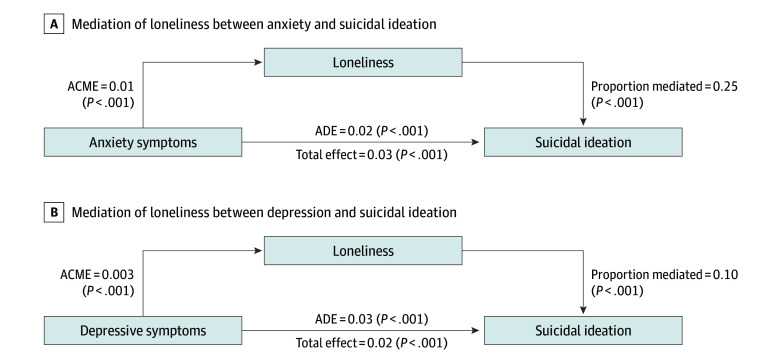
Mediational Model of the Role of Loneliness Between Anxiety Symptoms and Suicidal Ideation Loneliness was assessed using the UCLA Loneliness Scale, depressive symptoms were assessed using the first 8 items of the 9-item Patient Health Questionnaire (PHQ-9), anxiety symptoms were assessed using the 7-item Generalized Anxiety Disorder scale (GAD-7), and suicidal ideation was assessed using item 9 of the PHQ-9. A, Loneliness mediating the association between anxiety symptoms (GAD-7) and suicidal ideation (PHQ-9 item 9). B, Loneliness mediated depressive symptoms (PHQ-9 items 1-8) and suicidal ideation (item 9 of the PHQ-9). In both models, loneliness partially mediated the association with suicidal ideation. The direct associations of anxiety and depressive symptoms with suicidal ideation are still significant even after accounting for loneliness. This pattern suggests that the mediation is partial and not full. ACME indicates average causal mediation effect; ADE, average direct effect.

Figure 3 displays the mediational models wherein loneliness was tested as a mediator in the association between anxiety symptoms and suicidal ideation as well as depressive symptoms and suicidal ideation. All values reflect scores related to the self-report measures.

The total associations of both models were significant (anxiety symptoms model: total associations, 0.03; *P* < .001; depressive symptoms model: total associations, 0.02; *P* < .001) (Figure 3). Indirect associations in both models were also significant, indicating that loneliness mediated a significant amount of the variance in the associations between anxiety symptoms and suicidal ideation (ACME = 0.01; proportion of mediated associations, 0.25; *P* < .001) as well as depressive symptoms and suicidal ideation (ACME = 0.003; proportion of mediated associations, 0.10; *P* < .001). The direct associations of anxiety symptoms with suicidal ideation (ADE = 0.02; *P* < .001) as well as depressive symptoms with suicidal ideation (ADE = 0.03; *P* < .001) were also significant even after the addition of loneliness as a mediator.

The total associations, the proportion of mediated associations, and the ACMEs were larger in the model where loneliness mediates anxiety symptoms and suicidal ideation compared with the model where loneliness mediates depressive symptoms and suicidal ideation. However, in both cases, a significant amount of the associations of anxiety symptoms and depressive symptoms with suicidal ideation was mediated by loneliness. In brief, supplemental analyses indicated that loneliness remained a partial mediator in both models for both men and women. However, among transgender and nonbinary respondents, loneliness did not account for significant mediation between anxiety symptoms and suicidal ideation or depressive symptoms and suicidal ideation. The links between anxiety symptoms and suicidal ideation (eFigure 5 in Supplement 1) as well as depressive symptoms and suicidal ideation (eFigure 6 in Supplement 1) varied by age such that higher anxiety and depressive scores conferred more risk for suicidal ideation among younger participants.

## Discussion

Analyses were conducted using the All of Us Research Program to investigate the mediating role of loneliness between anxiety symptoms and suicidal ideation as well as depressive symptoms and suicidal ideation. Findings indicated that (1) anxiety symptoms, depressive symptoms, loneliness, and suicidal ideation were significantly and positively correlated; (2) anxiety symptoms, depressive symptoms, and loneliness independently explained significant variance in suicidal ideation; and (3) loneliness partially mediated the association between anxiety symptoms and suicidal ideation as well as depressive symptoms and suicidal ideation. These findings suggest the role of loneliness as a mediator between anxiety symptoms and suicidal ideation as well as depressive symptoms and suicidal ideation. They also suggest the potential use for a widescale, transdiagnostic approach to prevent or reduce loneliness to arrest some of the progression from anxiety and depressive symptoms toward suicidal ideation.

Regarding the first of these objectives, anxiety symptoms, depressive symptoms, and loneliness were significantly and positively correlated with suicidal ideation. This finding is consistent with both our hypothesis and previous research.^5,6,7,8,9,10,11,12,13,42,43^ Depressive symptoms, followed by anxiety symptoms, then loneliness, held the strongest correlation with suicidal ideation.

A similar pattern was evident regarding the second aim. When examining the simultaneous association of loneliness, anxiety symptoms, and depressive symptoms with suicidal ideation, all accounted for a significant portion of the variance. Depressive symptoms, followed by loneliness, then anxiety symptoms, accounted for the greatest share in suicidal ideation, with depressive symptoms accounting for nearly double the variance than loneliness.

Findings related to the third aim showed that loneliness partially mediated the association of anxiety symptoms and suicidal ideation as well as depressive symptoms and suicidal ideation. The associations of anxiety symptoms and depressive symptoms with suicidal ideation were in part mediated by loneliness.

This work extends previous work. Extant literature documented direct^9,10,11,12,13^ and indirect associations^28,29,30,31,32,33,34^ between anxiety symptoms and depressive symptoms with suicidal ideation. However, none of the previous studies investigated those direct and indirect associations in a single model. The present work places those direct and indirect associations into a single model, finding that when all constructs are included, loneliness partially mediates the association between anxiety symptoms and suicidal ideation as well as depressive symptoms and suicidal ideation. In these associations between anxiety symptoms and suicidal ideation as well as depressive symptoms and suicidal ideation, much of the associations are accounted for by loneliness.

These findings could point to 3 avenues to interrupt the development and/or intensification of suicidal ideation. Reducing depressive symptoms and anxiety symptoms are of course 2 avenues likely to arrest suicidal ideation. This has been a source of intervention research for decades, which has centered around mindfulness-based therapies, cognitive behavioral therapies, and pharmaceuticals (including ketamine) to reduce depression and/or anxiety in the hopes of ameliorating suicidal ideation.^44,45,46,47^ However, most of these interventions rely on licensed clinicians for delivery, which is not sustainable given nationwide shortages of qualified mental health clinicians and an abundance of barriers (eg, stigma and financial, geographical, and transportation barriers) to care.^48,49,50^ The third and perhaps more sustainable pathway could be to reduce loneliness, in effect buffering the associations of anxiety symptoms and depressive symptoms with suicidal ideation. This is likely a more scalable and person-centered approach in that people experiencing anxiety symptoms, depressive symptoms, and loneliness can engage with people and activities that are consistent with their values, cultures, goals, and preferences. Reduction of loneliness symptoms likely does not rely on access to licensed mental health clinicians, a scarce resource in today’s health care landscape.

The development of scalable interventions targeting loneliness is very challenging in the field of mental health today. Effective, scalable, low-cost interventions to reduce loneliness are not yet widely available, and a meta-analysis of digital technology interventions to reduce loneliness among older adults^51^ found that these tools are not yet developed. Analyses of 6 digital interventions aimed at reducing loneliness found that none of the interventions yielded statistically significant improvement in loneliness; furthermore, the quality of evidence was suboptimal. This lack of effective interventions to reduce loneliness may be a place for single-session interventions to be developed, which could serve as a primary intervention to prevent the onset of loneliness. Single-session interventions have demonstrated utility in the prevention of anxiety, depression, eating disorders, and substance use disorders.^52^ However, these brief intervention approaches encounter their own barriers, which include clinician hesitancy^53^ and lack of awareness among potential users.^54^

### Limitations

This study has some limitations, which relate primarily to the dataset in use. The All of Us Research Program is aimed at being a nationally representative dataset, yet it has not yet reached its full potential for findings to be generalized to the US population. At present, the dataset is composed primarily of White, non-Hispanic adults in the US who have self-selected into the dataset, representing only a small subset of the general US population. Furthermore, these data are cross-sectional, which prohibits us from fully assessing causality and temporal precedence. Although findings are interpreted in the direction of anxiety and depressive symptoms toward loneliness and finally suicidal ideation, this may not be the case. These findings rely on self-report surveys taken at a single point in time, which does not capture the changes in these phenomena that people experience within even a single day. Suicidal ideation was measured via a single item, which, again, does not capture the multifaceted and (for some patients) rapidly changing nature of the phenomenon.

## Conclusions

In this cross-sectional study of 62 685 US adults within the NIH All of Us Research Program, findings indicated that loneliness partially mediated the association between anxiety symptoms and suicidal ideation as well as depressive symptoms and suicidal ideation. Although anxiety and depressive symptoms were associated with suicidal ideation, much of these associations appeared to be mediated by loneliness. This finding highlights loneliness as a potential transdiagnostic treatment target that could be focused on via scalable interventions to reduce the incidence and intensity of suicidal ideation.

## Notes

Supplement 1. **eFigure 1.** Among Women, Findings Indicated That Loneliness Partially Mediates the Effects of Anxiety and Depressive Symptoms Towards Suicidal Ideation **eFigure 2.** Among Men, Findings Showed That Loneliness Once Again Partially Mediates the Effects of Anxiety and Depressive Symptoms Towards Suicidal Ideation **eFigure 3.** Among Nonbinary Participants, Direct Effects From Anxiety And Depressive Symptoms Are Significant; However, These Effects Are Not Driven Through Loneliness **eFigure 4.** Among Transgender Participants, Direct Effects From Anxiety Symptoms to Suicidal Ideation are Significant; However, These Effects Are Not Driven Through Loneliness **eFigure 5.** The Link Between Depressive Symptoms and Suicidal Ideation Varies by Age Such That Higher Depressive Scores Confer More Risk for Suicidal Ideation in Younger Participants **eFigure 6.** Anxiety Symptoms and Suicidal Ideation Have a Stronger Relation Among Younger Participants, Such That Anxiety Symptoms Confer Greater Risk of Suicidal Ideation in Younger, as Compared to Older, Participants

Supplement 2.
Data Sharing Statement
