# Supplementary material for: Loneliness, Anxiety Symptoms, Depressive Symptoms, and Suicidal Ideation in the All of Us Dataset
Source: JAMA Netw Open. 2026 Mar 4;9(3):e260596. doi: 10.1001/jamanetworkopen.2026.0596 (PMC12961520; doi:10.1001/jamanetworkopen.2026.0596)
Supplement: Supplement 1. — eFigure 1. Among Women, Findings Indicated That Loneliness Partially Mediates the Effects of Anxiety and Depressive Symptoms Towards Suicidal Ideation eFigure 2. Among Men, Findings Showed That Loneliness Once Again Partially Mediates the Effects of Anxiety and Depressive Symptoms Towards Suicidal Ideation eFigure 3. Among Nonbinary Participants, Direct Effects From Anxiety And Depressive Symptoms Are Significant; However, These Effects Are Not Driven Through Loneliness eFigure 4. Among Transgender Participants, Direct Effects From Anxiety Symptoms to Suicidal Ideation are Significant; However, These Effects Are Not Driven Through Loneliness eFigure 5. The Link Between Depressive Symptoms and Suicidal Ideation Varies by Age Such That Higher Depressive Scores Confer More Risk for Suicidal Ideation in Younger Participants eFigure 6. Anxiety Symptoms and Suicidal Ideation Have a Stronger Relation Among Younger Participants, Such That Anxiety Symptoms Confer Greater Risk of Suicidal Ideation in Younger, as Compared to Older, Participants [file jamanetwopen-e260596-s001.pdf]

Supplementary Online Content

Musacchio Schafer K, Franklin J, Embí PJ, Walsh CG. Loneliness, anxiety symptoms, depressive symptoms, and suicidal ideation in the All of Us dataset. *JAMA Netw Open.* 2026;9(3):e260596. doi:10.1001/jamanetworkopen.2026.0596

eFigure 1. Among Women, Findings Indicated That Loneliness Partially Mediates the Effects of Anxiety and Depressive Symptoms Towards Suicidal Ideation

eFigure 2. Among Men, Findings Showed That Loneliness Once Again Partially Mediates the Effects of Anxiety and Depressive Symptoms Towards Suicidal Ideation

eFigure 3. Among Nonbinary Participants, Direct Effects From Anxiety And Depressive Symptoms Are Significant; However, These Effects Are Not Driven Through Loneliness

eFigure 4. Among Transgender Participants, Direct Effects From Anxiety Symptoms to Suicidal Ideation are Significant; However, These Effects Are Not Driven Through Loneliness

eFigure 5. The Link Between Depressive Symptoms and Suicidal Ideation Varies by Age Such That Higher Depressive Scores Confer More Risk for Suicidal Ideation in Younger Participants

eFigure 6. Anxiety Symptoms and Suicidal Ideation Have a Stronger Relation Among Younger Participants, Such That Anxiety Symptoms Confer Greater Risk of Suicidal Ideation in Younger, as Compared to Older, Participants

This supplementary material has been provided by the authors to give readers additional information about their work.

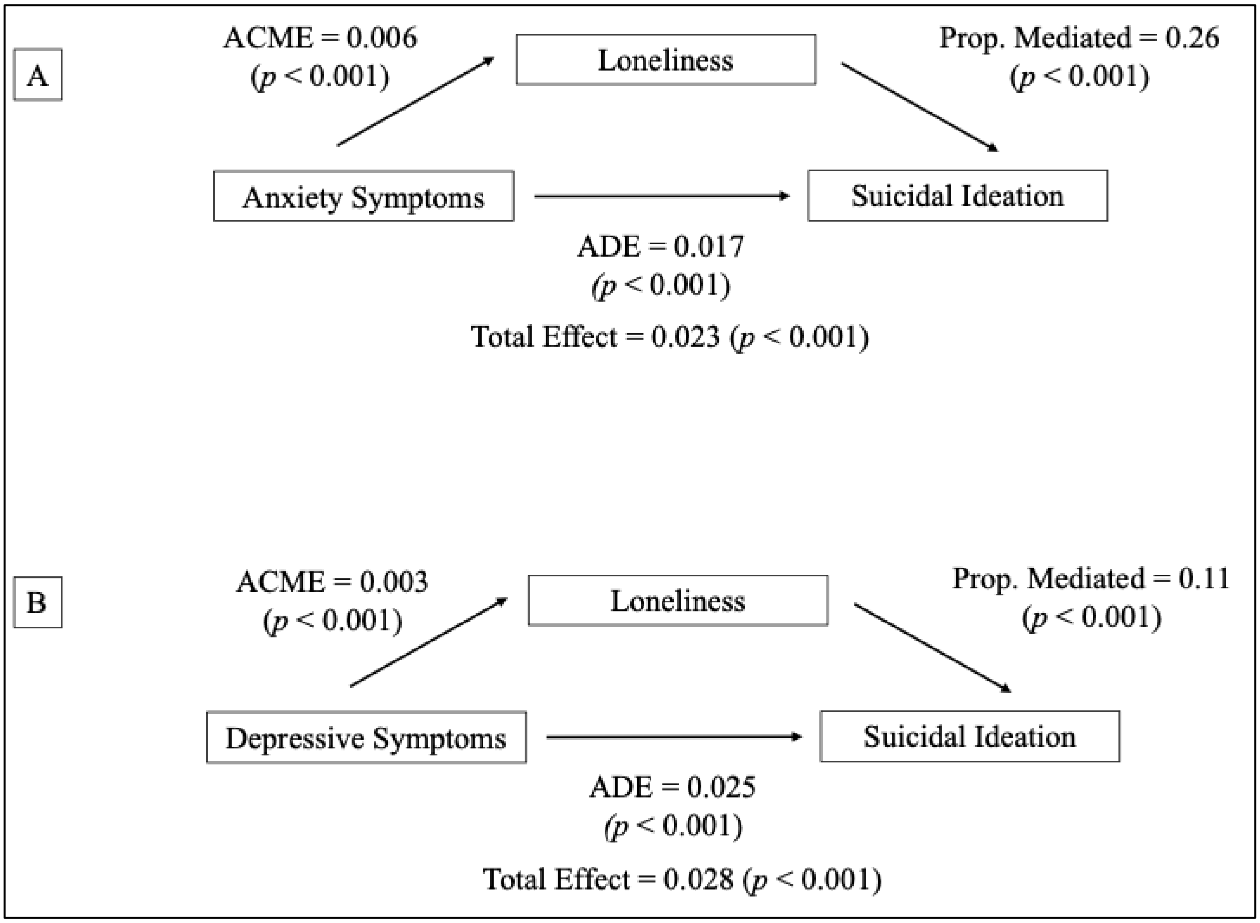

eFigure 1. Among Women, Findings Indicated That Loneliness Partially Mediates the Effects of Anxiety and Depressive Symptoms Towards Suicidal Ideation. Loneliness is a slightly stronger mediator in the link between anxiety symptoms and suicidal ideation as compared to the link between depressive symptoms and suicidal ideation.

© 2026 Musacchio Schafer K et al. *JAMA Network Open.*

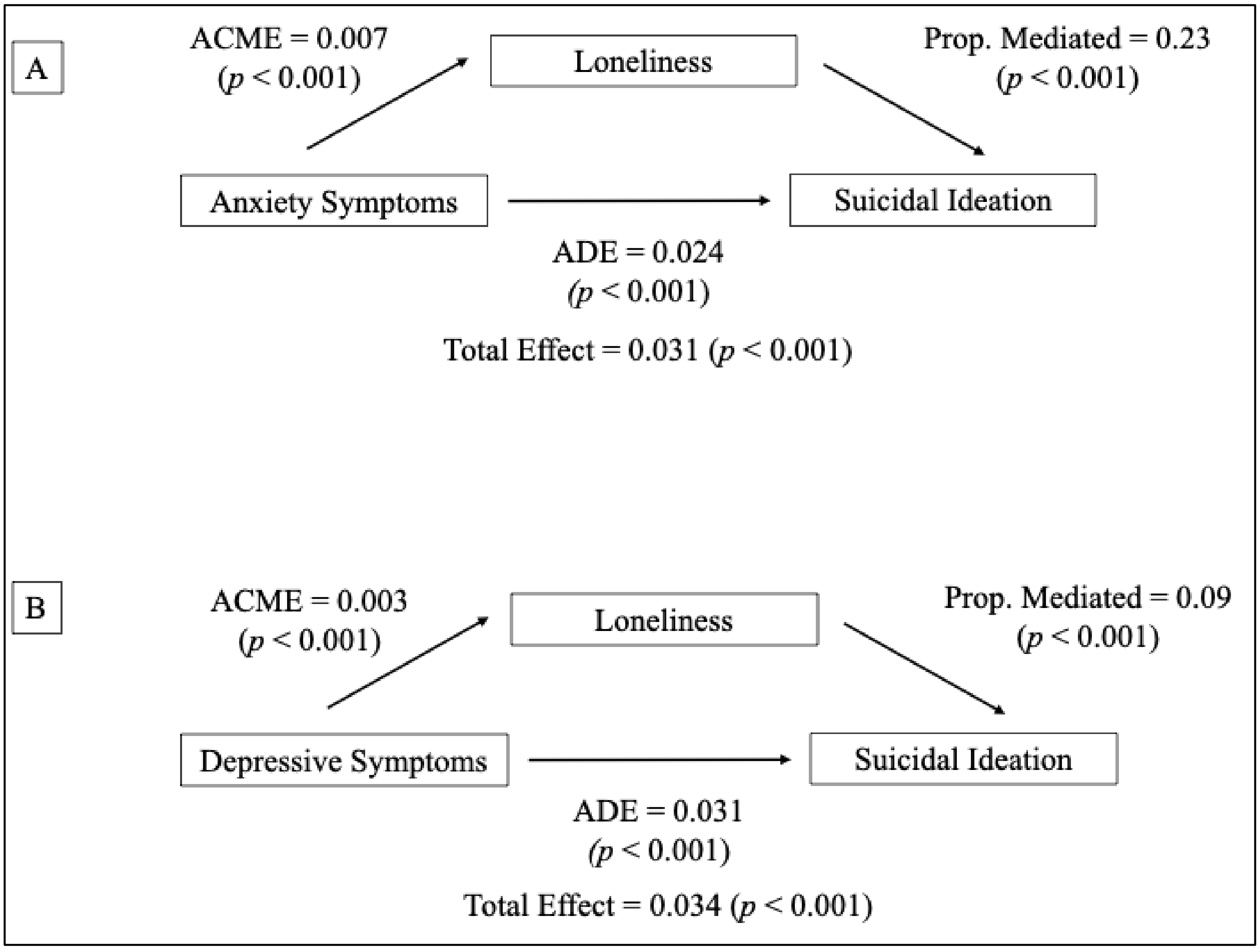

eFigure 2. Among Men, Findings Showed That Loneliness Once Again Partially Mediates the Effects of Anxiety and Depressive Symptoms Towards Suicidal Ideation. As was the case with the women only models, loneliness is a stronger mediator in the link between anxiety symptoms and suicidal ideation as compared to the link between depressive symptoms and suicidal ideation. The total effects of the models are stronger among men than for women.

© 2026 Musacchio Schafer K et al. *JAMA Network Open.*

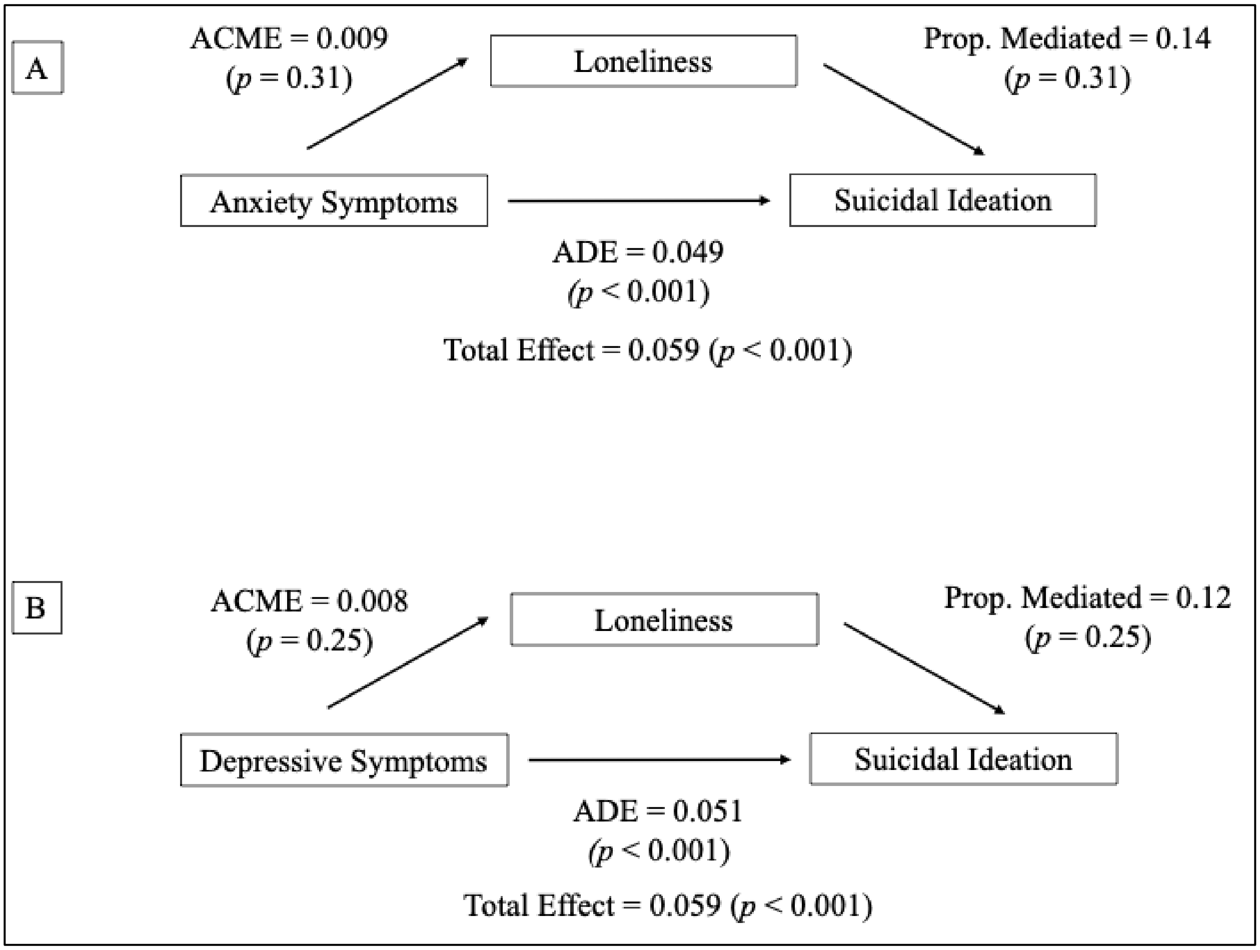

eFigure 3. Among Nonbinary Participants, Direct Effects From Anxiety And Depressive Symptoms Are Significant; However, These Effects Are Not Driven Through Loneliness. Loneliness does not mediate the link between anxiety and/or depressive symptoms with suicidal ideation.

© 2026 Musacchio Schafer K et al. *JAMA Network Open.*

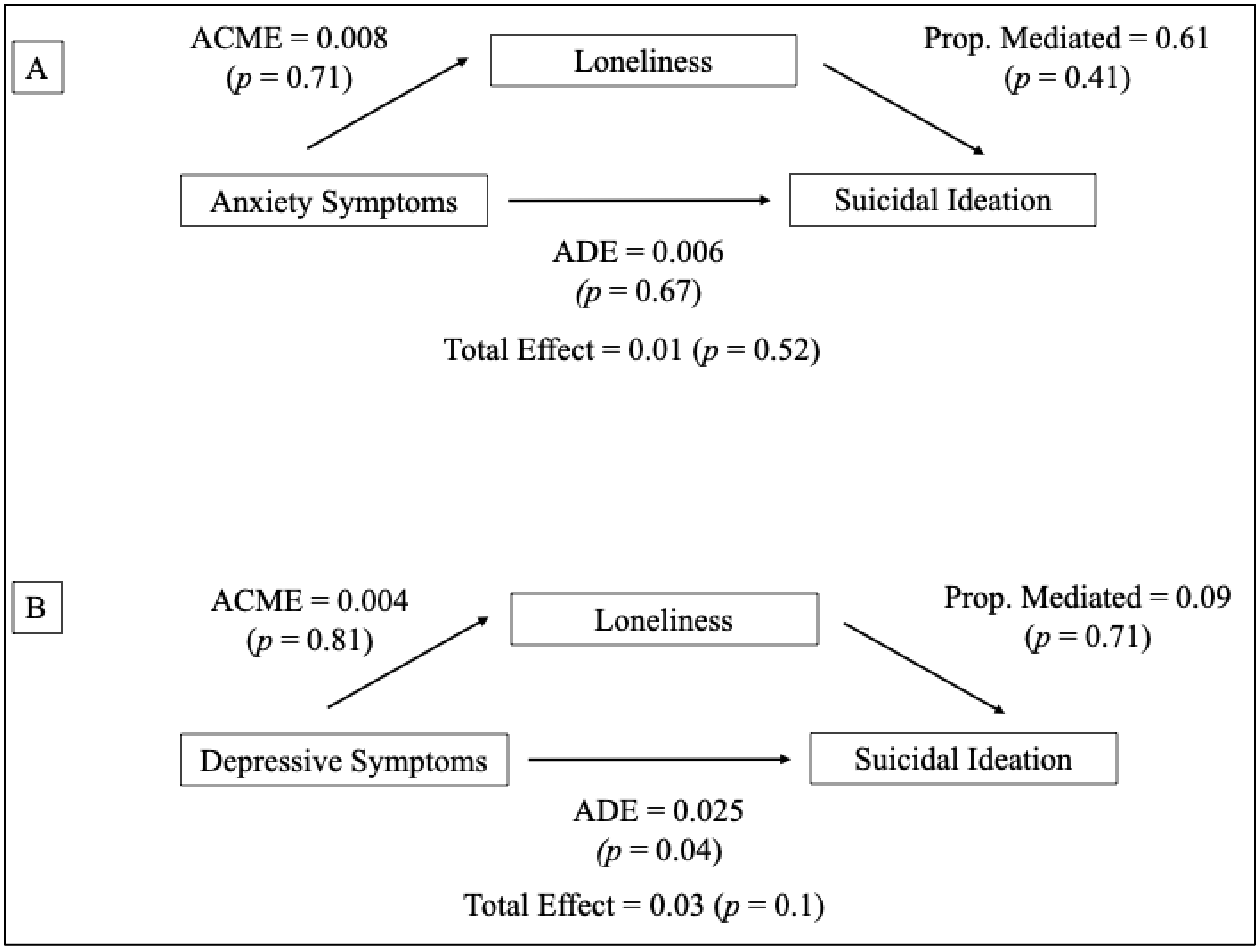

eFigure 4. Among Transgender Participants, Direct Effects From Anxiety Symptoms to Suicidal Ideation are Significant; However, These Effects Are Not Driven Through Loneliness. Effects from anxiety symptoms to suicidal ideation are not significant. In both models, loneliness does not mediate the link between anxiety and/or depressive symptoms with suicidal ideation.

© 2026 Musacchio Schafer K et al. *JAMA Network Open.*

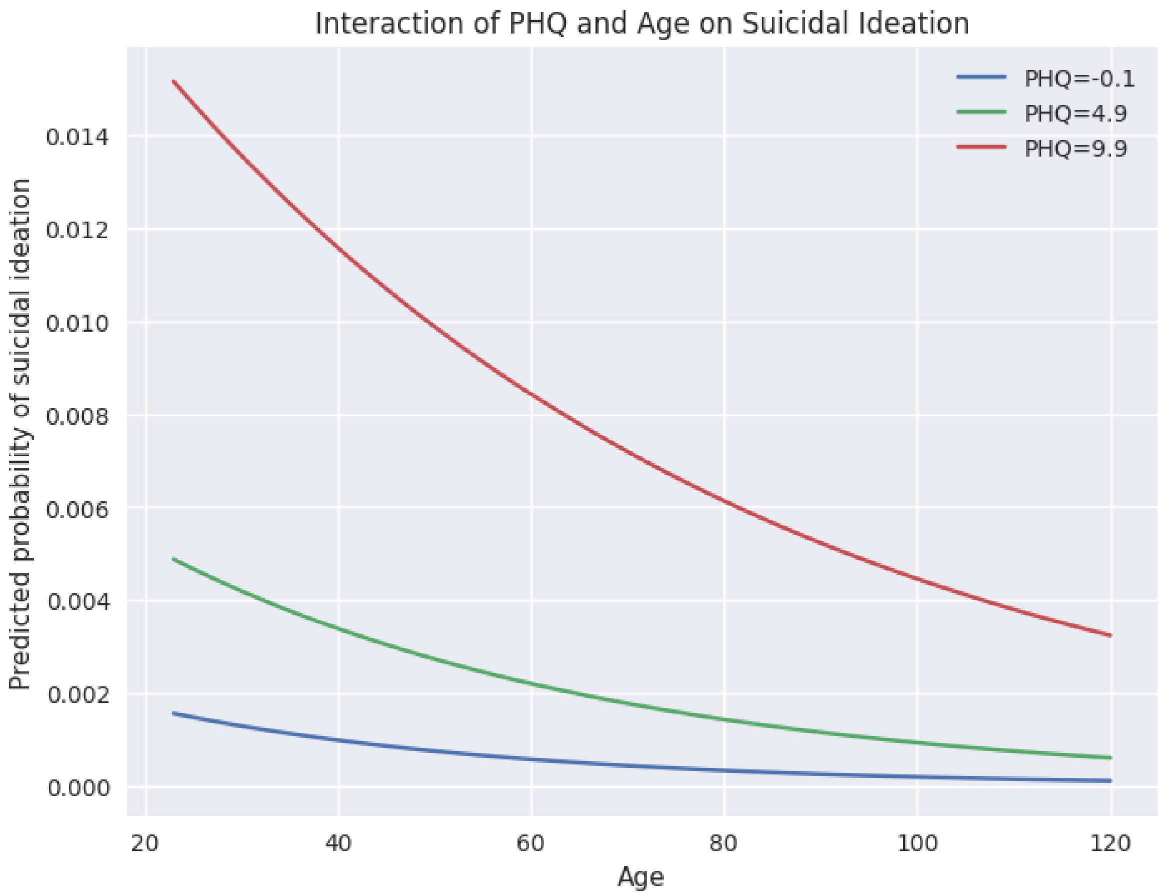

Variable	Coefficient	Std. Error	z-value	p-value	95% CI		Odds Ratio
Intercept	-5.139	0.366	-14.033	<0.001	-5.857	-4.421	0.0059
Anxiety Symptoms	0.1483	0.026	5.714	<0.001	0.097	0.199	1.16
Age	-0.0287	0.006	-4.456	<0.001	-0.041	-0.016	0.972
Anxiety x Age	0.0016	<0.001	3.396	0.001	0.001	0.003	1.002

eFigure 5. The Link Between Depressive Symptoms and Suicidal Ideation Varies by Age Such That Higher Depressive Scores Confer More Risk for Suicidal Ideation in Younger Participants

© 2026 Musacchio Schafer K et al. *JAMA Network Open.*

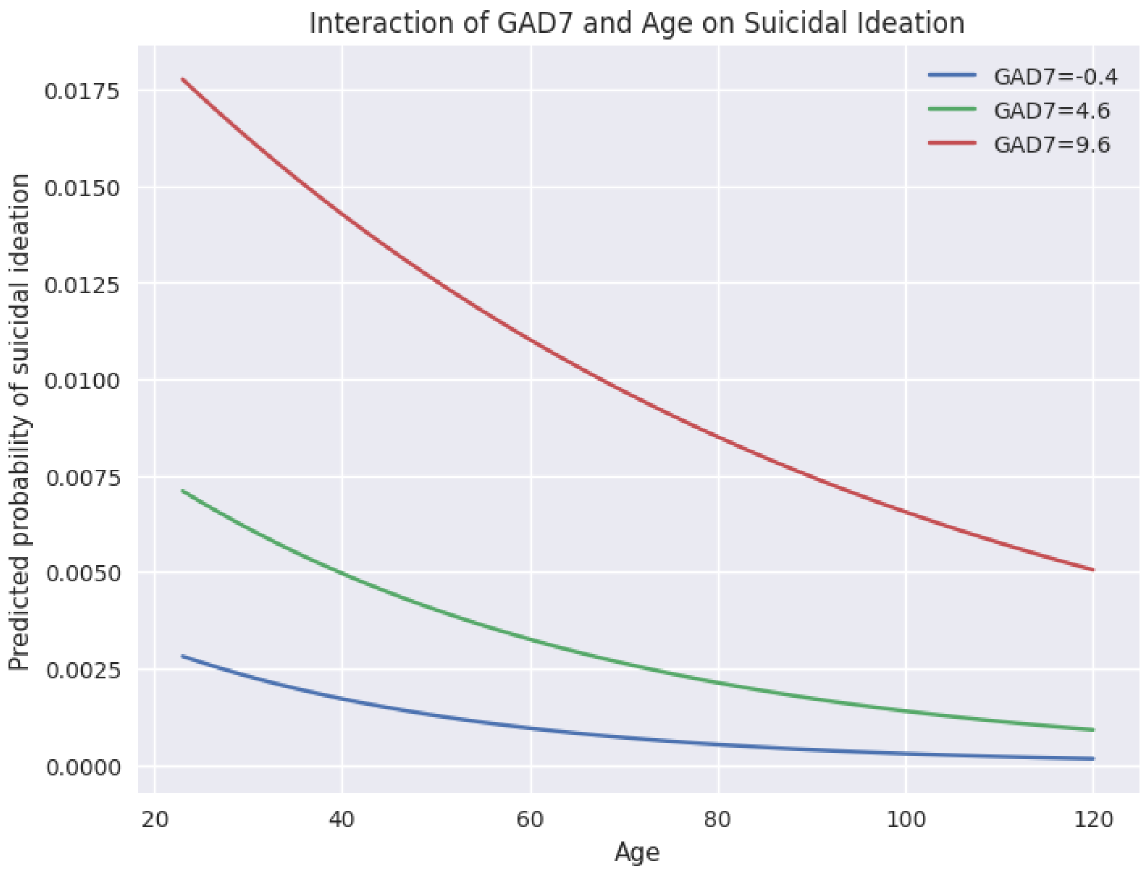

Variable	Coefficient	Std. Error	z-value	p-value	95% CI		Odds Ratio
Intercept	-5.8239	0.413	-14.1	<0.001	-6.633	-5.014	0.00295
Depressive Symptoms	0.2032	0.026	7.926	<0.001	0.153	0.253	1.225
Age	-0.0271	0.007	-3.682	<0.001	-0.041	-0.013	0.973
Depressive Symptoms x Age	0.0011	<0.001	2.368	0.018	<0.001	0.002	1.001

eFigure 6. Anxiety Symptoms and Suicidal Ideation Have a Stronger Relation Among Younger Participants, Such That Anxiety Symptoms Confer Greater Risk of Suicidal Ideation in Younger, as Compared to Older, Participants.

© 2026 Musacchio Schafer K et al. *JAMA Network Open.*
