# Supplementary material for: Loneliness, Anxiety Symptoms, Depressive Symptoms, and Suicidal Ideation in the All of Us Dataset
Source: JAMA Netw Open. 2026 Mar 4;9(3):e260596. doi: 10.1001/jamanetworkopen.2026.0596 (PMC12961520; doi:10.1001/jamanetworkopen.2026.0596)
Supplement: Supplement 2. — Data Sharing Statement [file jamanetwopen-e260596-s002.pdf]

Data Sharing Statement

Musacchio Schafer. Loneliness, Anxiety Symptoms, Depressive Symptoms, and Suicidal Ideation in the All of Us Dataset. *JAMA Netw Open*. Published March 04, 2026. doi:10.1001/jamanetworkopen.2026.0596

Data
Data available: Yes
Data types: Deidentified participant data
How to access data: All of Us Dataset is open available.
When available: With publication

Supporting Documents
Document types: None

Additional Information
Who can access the data: All of Us Dataset is open available.
Types of analyses: All of Us Dataset is open available.
Mechanisms of data availability: All of Us Dataset is open available.
